# Repurposing Amphotericin B: anti-microbial, molecular docking and molecular dynamics simulation studies suggest inhibition potential of Amphotericin B against MRSA

**DOI:** 10.1186/s13065-023-00980-9

**Published:** 2023-06-29

**Authors:** Neha Farid, Khair Bux, Kashif Ali, Asma Bashir, Rahima Tahir

**Affiliations:** Department of Biosciences, Faculty of Life Sciences, Shaheed Zulfikar Ali Bhutto Institute of Science and Technology (SZABIST), Karachi, Pakistan

**Keywords:** Anti-fungal, MRSA, Docking, Repurposing, Penicillin binding protein

## Abstract

Amphotericin B (AMPH) is an anti-fungal drug and this study, for the first time as best of our knowledge, reports the repurposing of the Amphotericin B. The drug was found to show significant antibacterial potential revealed by antimicrobial screening, molecular docking, and mode of action analysis targeting Penicillin Binding Protein 2a (PBP 2a protein) which is target of β-lactam drugs and is involved in cell wall synthesis. Mode of action analysis showed the drug to have hydrophobic and hydrophilic interactions with both C-terminal, trans-peptidase and non-penicillin binding domain of the protein. Additionally, to evaluate the impact of ligand binding on the protein's conformational dynamics, molecular dynamics (MD) simulations were used. Comparative Dynamical flexibility (RMSF) and Dynamics Cross Correlation (DCCM) followed by MD simulations revealed the complex formation significantly effecting structural dynamics of the enzyme significantly in the non-penicillin binding domain (327–668) and slightly in trans peptidase domain. Radius of gyration assessment further showed ligand binding also decreasing over all compactness of protein. Secondary structure analysis indicated the complex formation changing the conformational integrity in non-penicillin binding domain. Hydrogen bond analysis and MMPBSA, free energy of calculations followed by MD simulations, also complemented the antimicrobial and molecular docking revelations suggesting Amphotericin B to have substantial antibacterial potential.

## Introduction

**Fig. 1:**
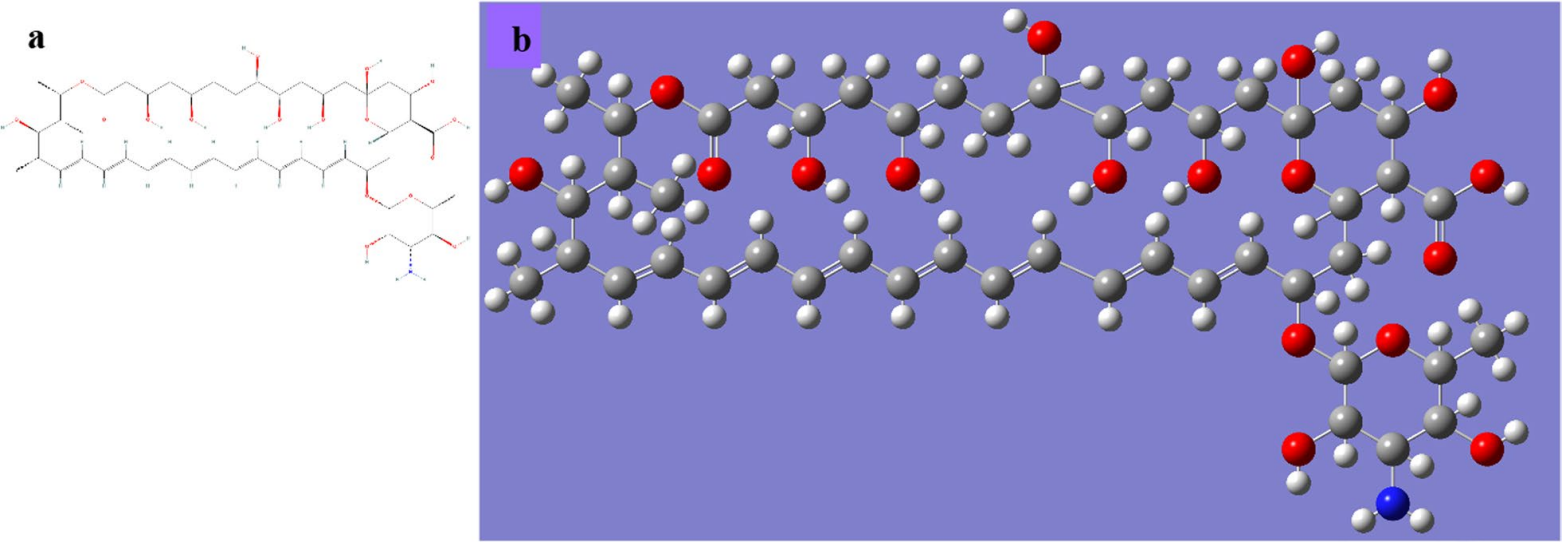
**a** 2D representation of the Amphotericin B **b** 3-D representation of ligand after Quantum Mechanically geometry optimization

*Streptomyces nodosus produces the antifungal polyene macrolide known as amphotericin B.* (Fig. 1) [1–3]. By attaching to a sterol spot on the membrane, it alters membrane permeability and causes the death of fungal cells. It is discovered to be effective against infections caused by Aspergillus fumigatus, Candida albicans, Histoplasma capsulatum, Coccidioides immitis, and Cryptococcus neoformans. Amphotericin B is only used in cases of life-threatening infections because of its severe toxicity [3, 4].

It is known that the medication alters the permeability of the cell membrane of the host cell.

By producing a barrel-like shape, the drug creates gaps in the cell membrane, resulting in the irregular movement of ions and metabolites within and outside the cell [5, 6]. This uneven transit of components within the cell causes harm to the integrity of the cell, which ultimately results in cell death. Amphotericin B is discovered to interact more strongly with sterols in the cell membrane. Amphotericin B monomers are necessary for modifying cellular permeability in a specific amount [6–8].

A Gram-positive microbe called MRSA or Methicillin Resistant *Staphylococcus aureus* is known to cause severe skin infections such as inflammation causing red pimples, septicemia and food poisoning. It has become a bacterium of interest, as it is resistant to many pharmaceutical drugs such as Penicillin, β- Lactams that includes Amoxicillin and Methicillin. MRSA is *Staphylococcus aureus*, which has the mecA gene, is found to be resistant to methicillin and other [9–16].β-lactamase production plays a major role in development of antibiotic resistance in both gram-positive and gram- negative pathogens by degrading the β-lactam antibiotics. Microorganisms inhibit the bactericidal effect of the antibiotics by many biochemical phenomena. The mechanisms include enzyme-inactivating antibiotics, mutational or post-translational changes in the binding sites of the antibiotic targets making them ineffective and reduced retention of the antibiotics in the bacterial cell by the action of the efflux pumps [17–22].β-Lactam antibiotics have been found to interfere with synthesis of peptidoglycan by inhibiting the action of enzymes known as penicillin-binding proteins (PBPs) [22–24]. Penicillin-binding proteins (PBPs) are the membrane-associated proteins which are involved in the synthesis of the main component of bacterial cell wall i.e. peptidoglycan. PBP have binding affinity to the antibiotic penicillin, which belongs to β-lactam. PBPs have vast importance as it has diverse functions in protein–protein interactions, antibiotic resistance, cell wall synthesis and many other regulatory functions [24–28].

As the use of existing medications for new indications (drug repurposing) is an efficient strategy not just for reducing drug development time and costs, but also for developing treatments for new diseases, even rare ones.

There are more than forty docking programmes now available, with AutoDock being the most popular. Identification of the binding mode is the goal of molecular docking, which calls for a search strategy that mimics native protein–ligand interactions [29]. The LGA, or Lamarckian Genetic Algorithm, is a novel approach that integrates the Genetic Algorithm (GA), Monte Carlo simulation, and a hybrid local search GA [30–32]. Using this approach, it becomes possible to thoroughly explore the conformations of protein–ligand complexes in search of the configurations with the most favorable binding energies [33]. The progress made in bioinformatics and computational biology has led to various new developments, including the ability to calculate inhibitory constants for docked conformations.

Molecular dynamics (MD) simulation is an indispensable tool for the study of macromolecules such as nucleosomes [34], ribosomes [35], membrane proteins, organic solids, proteins-ligand complexes, etc. Thanks to the improvements in force fields that came about through the progress of quantum physics and computational chemistry, the field of protein–ligand docking has undergone significant advancements in the last 40 years [36–39]. The use of simulation is widespread in investigating the connection between the structure and function of proteins and protein–ligand complexes.

With the right system configurations and the aid of high-speed supercomputers, generation of molecular dynamics can simulate the behavior of up to 500,000 atoms on a scale of nanoseconds [40]. Through the use of appropriate system configurations and high-speed supercomputers, molecular dynamics generation enables the simulation of actual biological systems, with the potential to model up to 500,000 atoms and their behavior on a nanosecond scale. However, due to the need to simulate intra- and interatomic interactions simultaneously, this requires thousands to millions of computational steps, making supercomputers necessary. Given that the structural and functional characteristics of biomolecules are linked to timeframes in the nanosecond and microsecond ranges, it is important to analyze the simulation on the shortest timescale possible, ideally in femtoseconds [41]. The OPLS (Optimized Potentials for Liquid Simulations) force field, which was created at Purdue University, shares many similarities with AMBER [42]. The OPLS3 force field is known for its higher accuracy in fitting torsional parameters when compared to other small molecule force fields. Molecular dynamics simulation is a valuable tool in exploring the conformational stability, dynamics, fluctuations, and deviations from the reference structure of proteins and protein–ligand complexes at various nanosecond intervals, as mentioned in the study [42].

Amphotericin B is effective against a wide range of fungal species, including *Cryptococcus neoformans, Candida albicans, Sporotrichum, Blastomyces dermatitidis, Histoplasma capsulatum, Coccidioides immitis,* and *Aspergillus fumigatus*. As a result, it is utilized in the treatment of a variety of fungal infections, including disseminated candidiasis, cryptococcosis, coccidioidomycosis, histoplasmosis, North American blastomycosis, aspergillosis, and sporotrichosis [2, 43]. When the usual formulation of Amphotericin B is contraindicated due to toxicity, particularly nephrotoxicity, lipid versions are less toxic and advised [44].

Amphotericin B is typically administered through intravenous or topical routes, although it can also be given via other methods such as oral lozenges, nebulized solution, intrathecal injection, and bladder irrigation [44]. As the use of existing medications for new indications (drug repurposing) is an efficient strategy not just for reducing drug development time and costs, but also for developing treatments for new diseases, even rare ones. In order to discover novel indications, several combined studies have been reported [45–48].

The current study as best of our knowledge also reports the repurposing of antifungal drug, Amphotericin B (AMPH), against Methicillin Resistant *Staphylococcus aureus* (MRSA). In the current study, antimicrobial effect of the AMPH against clinical strains of MRSA was investigated. Antimicrobial screening was followed by molecular docking studies targeting cell wall synthesizing proteins, penicillin-binding proteins (PBPs)^®^ for investigating the mode of action of ligand, AMPH against protein target at atomistic level. As Structural and dynamical insights give more understanding of changes in protein structure after complex formation. Thus, Molecular dynamics (MD) simulations was also carried to further explore the inhibition phenomenon of ligand in terms of structural and dynamical effects upon complexation. To evaluate the binding energy of simulated coordinates, the Molecular Mechanics Poisson-Boltzmann Surface Area (MMPBSA) approach was utilized. Additionally, the study explores potential future applications of the current findings.

## Materials and method

### Antimicrobial studies

Clinical sources provided five strains of Methicillin Resistant Staphylococcus aureus (MRSA) that were found to be resistant to multiple drugs. The colonies were further confirmed by the appearance of characteristic morphology of the *Staphylococcus aureus* colonies on Mannitol Salt Agar, Gram positive staining, positive results for the biochemical tests of Catalase, Coagulase and Hemolysis tests. The methicillin resistance was confirmed by the disk diffusion test of methicillin antibiotic (30 µg) against the obtained strains as per the guidelines of the Clinical Laboratory Standards Institute (CLSI).

### Compound susceptibility assays by MIC and MBC determination

Amphotericin B was purchased from Sigma-Aldrich and Oxoid. Amphotericin B was used in the concentrations ranging from 32 μg/ml to 0.5 μg/ml. MIC determination was done by the two-fold serial dilution tube test method. The bacterial culture suspensions were adjusted to 0.5 McFarland Turbidity standard. Experiments were conducted in replicates of three. Results for MIC were determined by observing the growth visually in the tubes after 18 h of incubation. For MBC Determination, 100 µl of suspension from each tube was spread-plated on the MSA and incubated for 18 h. Concentrations of drug which completely killed the bacteria by showing no growth after plating was considered as the MBC value, and the concentration lower than MBC was considered as the MIC value.

### Molecular docking

Amphotericin B's crystal structure was obtained from Pubchem (https://pubchem.ncbi.nlm.nih.gov/compound/Amphotericin). The recovered structure was further quantum mechanically optimized using the 6-31G* DFT approach and B3LYP level of theory. As shown in Fig. 1, the optimized structure is displayed. In order to perform the rigid docking, the ligands' rotatable bonds were considered non-rotatable. Before docking, Gasteiger charge calculation method (Gasteiger and Marsili, 1980) was utilized to assign partial charges to the ligand atoms. The study utilized the crystal structures of Penicillin Binding protein 1VQQ [49], were obtained from the RCSB Protein Data Bank (PDB) (http://www.rcsb.org/pdb/home/home.do). The PyMOL molecular graphic system, version 1.5.0.3 (www.pymol.org), was utilized to eliminate all hetero atoms and water molecules from the crystal structures. The binding site residues of the proteins were identified using previous research data [50].

Proteins were docked with Amphotericin B and experimental control inhibitors. The Grid box parameters were used setting up the grid parameters with spacing of Grid was set to 0.375 Å (default). Center grid box values were −11.432, −9.16, and 2.636. The points for grid numbers in accordance with the x, y, and z dimensions was set to be 60, 60 and 58. There were 431,893 total grid points on each map. The full 3-dimensional active site of the receptor was covered by these characteristics using Auto Dock Tools (ADT), a free graphic user interface of MGL software packages. The molecular docking program AutoDock4.2 [51, 52] was used to perform the docking experiment. The docking process utilized the Lamarckian Genetic Algorithm with a population size of 150 individuals to search for the ideal conformational space for the ligand. The maximum number of generations and evaluations were set to 27,000 and 2,500,000, respectively, while some parameters were left at their default values. (Table 1).

**Table 1:** Detailed picture of interaction of Amphotericin B with PBP2a

Compound	RMSD	Binding energy Kcal/Mol	Inhibition constant (Ki) uM	H-bonding forming residues	Amino acids involved in interactions
Amphotericin B	1.18	−9.01	84.66	ASP81	ASP81,GLU58,ILE102, ILE86

### Docking validation

Utilizing two different techniques, the docking approach was validated. Using AutoDock 4.2.6, the reported complex of Penicillin Binding Protein with ceftaroline (PDB ID3ZG0) was taken out and docked back into the active site (R). Manually, the co-crystallized complex was opened in a notepad, the inhibitor heteroatoms 6from the protein were removed, and the protein was then pasted into a new notepad and saved as an inhibitor in PDB file format. The method followed the same protocol, including the grid parameters. This was done in order to make sure that the inhibitor binds precisely to the active site cleft and must exhibit less deviation from the co-crystallized complex. The root mean square deviation (RMSD) was then computed by superimposing the re-docked complex with PyMOL 2.3^®^ on top of the reference co-crystallized complex. This was done to assure the validation of docking and to validate the docking technique.

### Molecular dynamics simulations

The structural complex of AMPH with penicillin-binding proteins (PBPs) and Apo protein was provided as the initial input for Gromacs version 5 MD simulations [53]. The OPLS-AA force field was employed to simulate the protein and ligand models [54–56]. Then, the protein–ligand complexes were subsequently solvated in a 14 Å solvent box containing SPC [57] (simple point charge) 94,947 water molecules. Entire charge of the systems was neutralized by adding 2 CL ions. Particle Mesh Ewald (PME) [58, 59] summation was utilized to derive the long-range electrostatic interactions. For covalent bond constraints, the Linear Constraint Solver (LINCS) [60] algorithm was applied. Several energy minimization steps were performed to stabilize the systems. Next, the systems were equilibrated at a temperature of 300 K for 100 ps using the NVT ensemble (constant Number of particles, Volume, and Temperature) followed by another 100 ps using the NPT ensemble (constant Number of particles, Pressure, and Temperature). Finally, the equilibrated systems were subjected to a 20 ns simulation with 2 fs time steps. The resulting MD trajectories were0 analyzed for further insights.

### The molecular mechanics/Poisson–Boltzmann surface area (MM-PBSA) approach was utilized to analyze the binding energy

The interaction free energies of each PBB-AMPH complex were determined using the MM/PBSA technique, which is a quantitative calculation of the binding free energy used to examine biomolecular complexes in the final stages of drug discovery [61]. In this study, the binding free energies were calculated using the last 1000 ps of the MD trajectories. To determine the binding free energies, the following set of equations was used:

1 $${\Delta G}_{bind}={G}_{complex}-\left({G}_{protein}+{G}_{ligand}\right)$$

2 $${\Delta G}_{bind}={\Delta E}_{MM}-T\Delta S+{T\Delta S}_{sol}$$

3 $${\Delta E}_{MM}={\Delta E}_{ele}+{\Delta E}_{vdw}$$

4 $${G}_{sol}={G}_{pol}+{G}_{nonpol}$$

5 $${G}_{nonpol}=g\Delta SASA+b$$

**Fig. 2:**
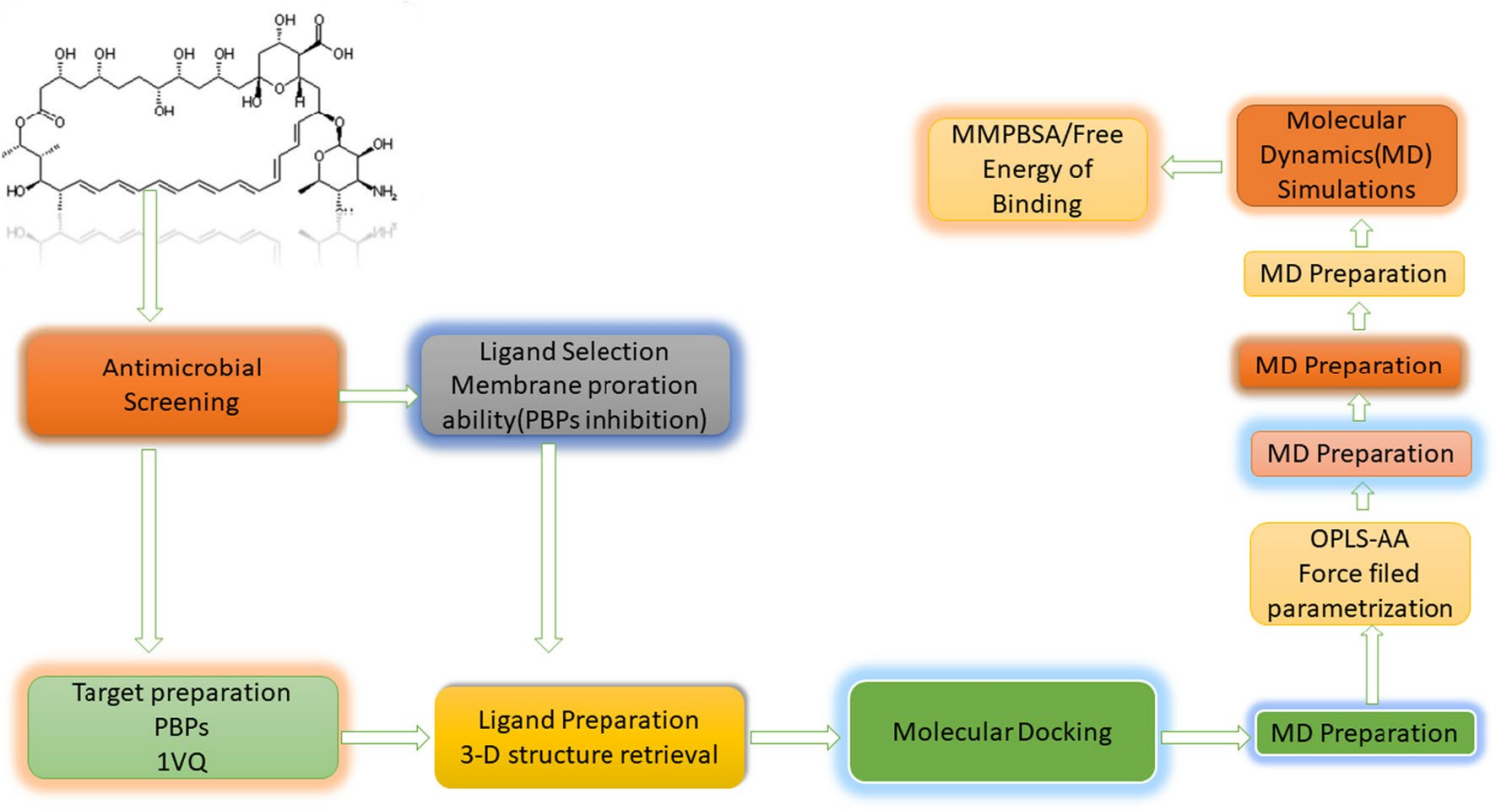
Working scheme of the current study

The binding free energies were calculated in this study using the last 1000 ps of the MD trajectories with the following equations. The total free energy of the protein–ligand complex, Gcomplex, was computed in Eq. (1), while Gprotein and Gligand represented the total free energies of the protein and ligand in solvent. The total binding energy, which included de-solvation of the ligand and the unbound protein, was calculated using Molecular Mechanics (MM) force-field parameters. Equations (2) and (3) determined the average potential energy of molecular mechanics in a vacuum (DEMM) and the solute entropic contribution at temperature T (Kelvin) (TDS), respectively. The solvation free energy (Gsol) was determined using Eq. (4), which included the sum of the electrostatic and non-electrostatic solvation energies (Gpol and Gnonpol, respectively). The polar solvation energy was determined by solving the Poisson-Boltzmann linear equation, while the nonpolar solvation energies were determined by calculating the solvent accessible surface area (SASA). In Eq. (5), c represented a coefficient of surface tension, while b is a fitting parameter [62]. Figure 2 represents the schematic plan of the study done.

## Results and discussions

### Antimicrobial studies

**Fig. 3:**
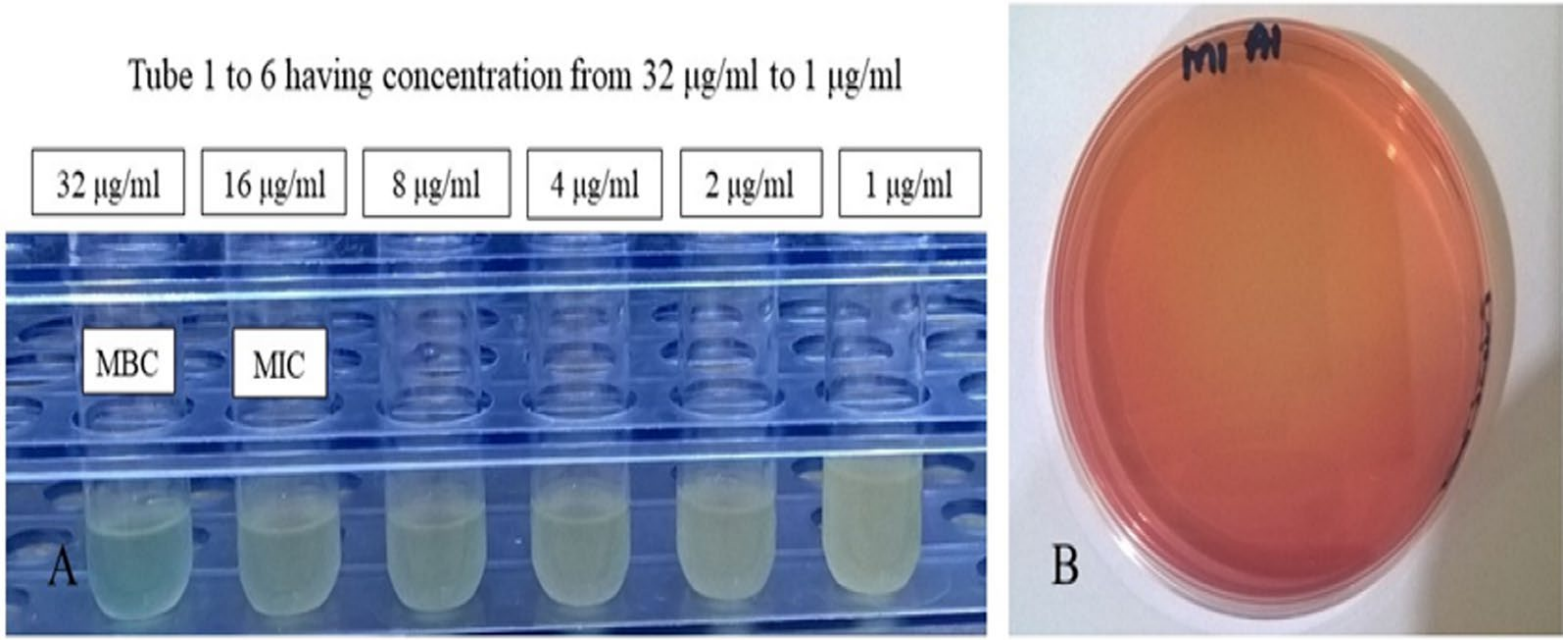
**a** Tubes showing MIC and MBC results, the amount of turbidity is increased by the bacterial growth as the concentration reduces. **b** Plate streaked with solution of tube having 32 μg/ml of Amphotericin B showed no bacterial growth

Amphotericin B is a well-known antifungal drug with outstanding activity against fungal infections. However, previous research indicates that the antifungal antibiotic is less efficient against bacterial illnesses. It has been proven to be somewhat effective against specific *Escherichia coli* strains. Its mechanism of action is aimed to disrupt cellular integrity, resulting in eventual cell death. Interestingly, Amphotericin B shown efficacy against Methicillin-Resistant *Staphylococcus aureus* strains in this study. Amphotericin B was utilised at concentrations ranging from 32 µg/ml to 0.5 µg/ml. For the Minimal Inhibitory Concentration Test, the medication was serially diluted with an initial concentration of 32 µg/ml. At a concentration of 32 µg/ml, the tubes were completely transparent, indicating the absence of bacterial growth. In the remaining tubes, the amount of turbidity rose as the antibiotic concentration moved towards the lower level (Fig. 3a). This similar pattern of growth suppression at 32 µg/ml Amphotericin B was observed across all strains. The tubes with no or minimal turbidity were further evaluated for the Minimum Bactericidal Concentration, and it was noted that the growth of bacteria was totally prevented at 32 µg/ml, and very little growth was observed at 16 µg/ml (Fig. 3b). Observed results led to the conclusion that 32 µg/ml of Amphotericin B functioned as the Minimum Bactericidal Concentration and 16 µg/ml as the Minimum Inhibitory Concentration for all five MRSA strains as shown in Table 2. These findings led to the novel conclusion that Amphotericin B can be used as an efficient antibiotic to treat fatal infections caused by MRSA, a multidrug-resistant bacteria.

**Table 2:** Results of Amphotericin B against the MRSA Strains

Amphotericin B					
Concentration (μg/ml)	MRSA 01	MRSA 02	MRSA 03	MRSA 04	MRSA 05
32	100	100	100	100	100
16	75	75	50	75	50
8	25	25	20	25	25
4	0	0	0	0	0
2	0	0	0	0	0
1	0	0	0	0	0
0.5	0	0	0	0	0

The number represents the percentage of bactericidal activity of the drug. 32 μg/ml shows 100% effective results as compared to the other concentrations

^*^100 represents complete inhibition of growth of bacterial cells; 0 represents full growth of bacterial cells

### Molecular docking

The Penicillin binding protein 2a has two domains, a C-terminal trans peptidase domain (residues 327–668) with similar overall fold with other trans-peptidases and the serine β-lactamases containing active site residues [63, 64] and a non-penicillin-binding allosteric domain (residues 27–326) [49]. Studies report a closed active site in C-terminal trans-peptidase domain, that substrate could not freely gain access to the active site and can explain bacteria resistance to antibiotic. PBP2a efficaciously recognises -lactam antibiotics as possible inhibitors, prefers the peptidoglycan substrate, and carries out the peptidoglycan-crosslinking process under physiological conditions [49]. β-lactams connect either particularly to the active site (covalently) [49] or to both the allosteric (non-covalent) and the active sites (covalent) [65]. An allosteric site in a non-penicillin-binding domain, distal from the active site is responsible to discriminate when properly occupied simultaneously opens the gatekeeper residues (Met641 and Tyr446) within the active site. These open or partially open form of active site confirming that allosteric site occupancy is the first step in the activation process of PBP2a [66, 67]_._

**Fig. 4:**
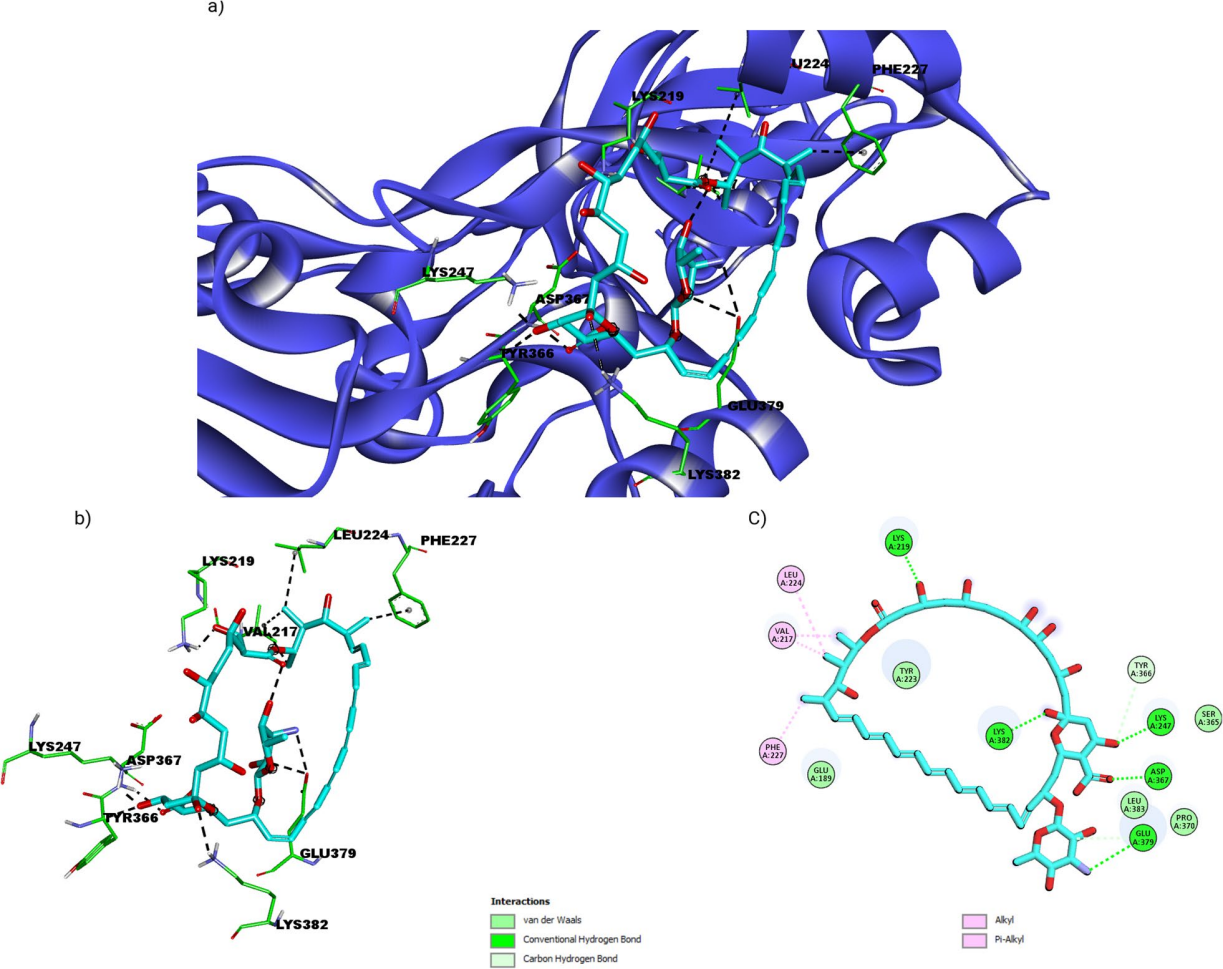
Depiction of docked pose of Amphotericin B with protein representation scheme: protein blue colored new ribbons, ligand cyan in stick and interacting amino acid residues are shown as stick in green color **a** shows full 3-D view of the ligand with protein amino acids residues **b** focused 3-D view of ligand with interacting amino acids residues **c** 2-D depiction of ligand and interacting residues with possible types intermolecular interactions supporting ligand binding

Our docking outputs explained the highest binding affinity of the ligand with the wild type (−7.623 kcal/mol), with inhibition constant 2.56uM and internal molecular energy about −8.56 kcal/mol. Molecular docking results revealed Amphotericin B (AMPH) to be actively binding with both active site C-terminal trans peptidase domain forming hydrogen bonding interactions with TYR 366 and Glu 377 and non-penicillin binding allosteric region forming hydrophobic and pi-pi interactions with allosteric region residues like LYS 219, LYS 382, LYS 247, ASP 367 and Glu 379 (Fig. 4). Therefore, binding mode of Amphotericin B revealed by docking results suggests compound to have reasonable inhibition potential against PBP2a.

### Docking validation

**Fig. 5:**
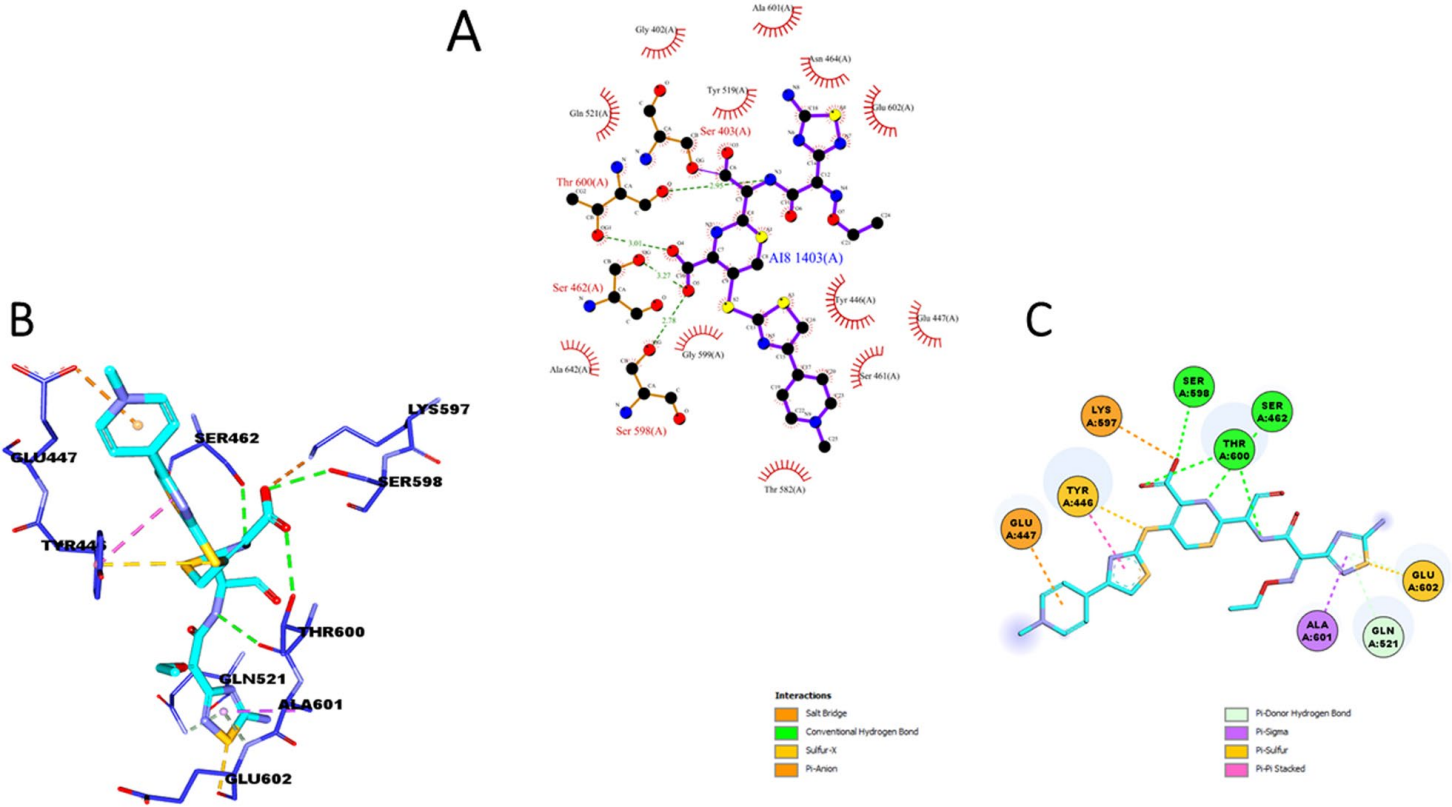
Docking validation **A** 2-D view of interaction of protein amino acid residues with drug in reported complexed crystal **B** 3-D view of red-cocked complex showing interactions of protein residues (stick blue) drug(cyan). **C** 2-D view of re-docked complex showing amino acid residues (circled) interacting with drug (cyan)

Molecular docking was validated through redocking the reported complex of PBBs with ceftaroline. As shown in Fig. 5 ligand was found to have interactions exactly with the same residues that were reported in complexed crystal. Moreover, estimated root mean square deviation (RMSD) by means of superimposition of re-docked complex was also found favorable [65].

### Molecular dynamics simulations

**Fig. 6:**
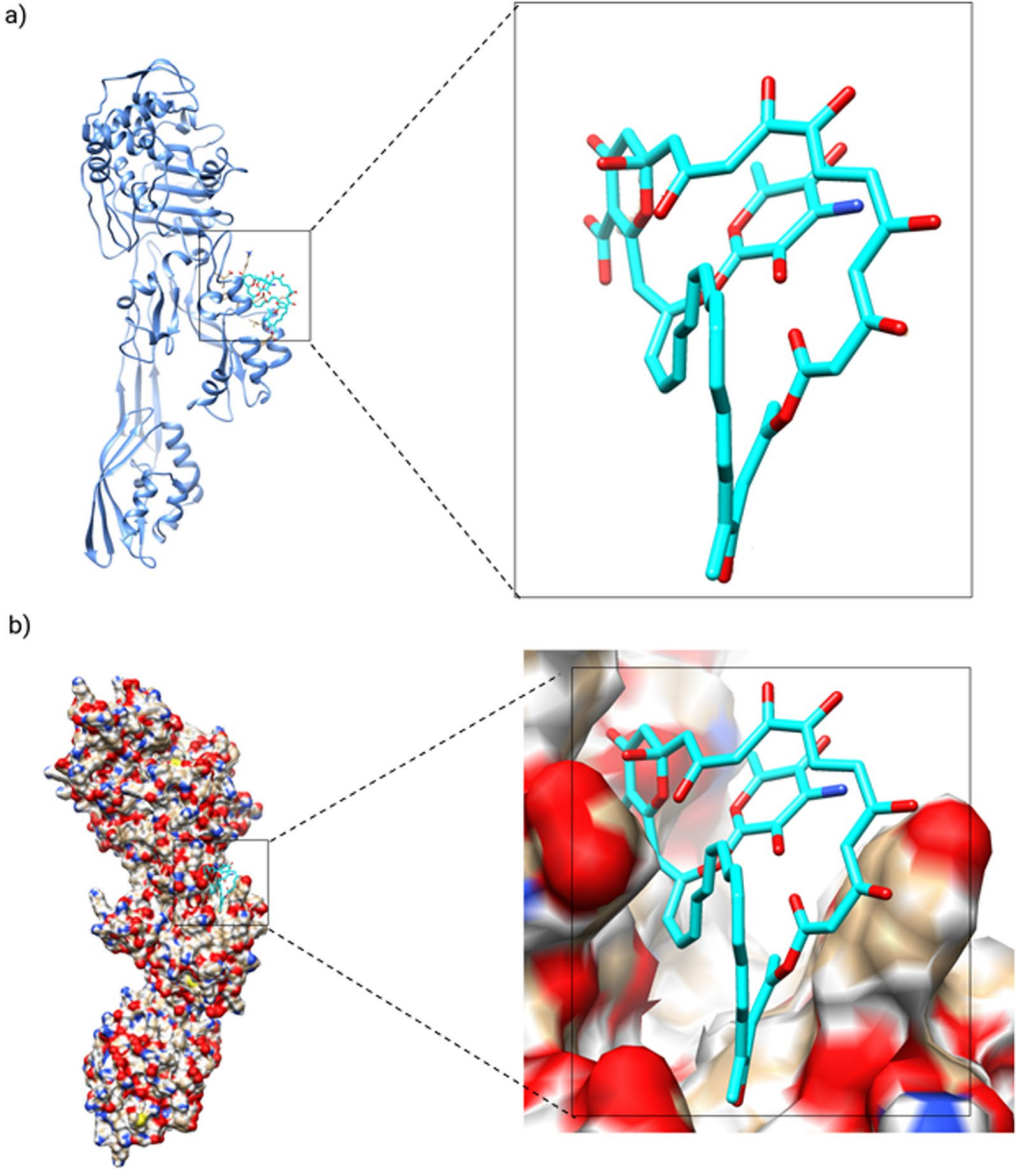
Representation of simulated complex **a** Full 3-D view of binding of ligand (stick in cyan color) with protein (blue colored new ribbons) **b** Surface depiction of the protein (blue color represents Nitrogen, green is for Oxygen, cyan, Carbon and white is for Hydrogen) showing binding cavity of bound ligand in simulated complex

**Fig. 7:**
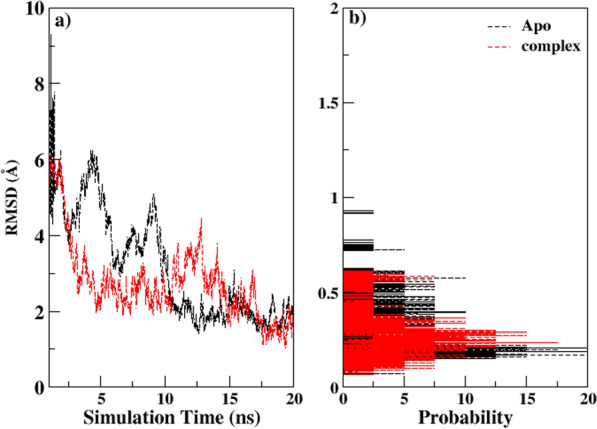
The root mean square deviation (RMSD) was plotted **a** as a function of simulation time and **b** as a probability plot, with the unbound protein represented in black and the complex in red

Using simulations of molecular dynamics, the PBPs enzyme and its complex with AMPH were studied. The stability of the three systems was assessed by computing the root mean square deviation (RMSD) of the heavy atoms of the enzyme over the course of the full trajectory run, as shown in Fig. 6. Figure 7 presents the RMSD plot for the enzyme without the ligand, which fluctuates around an average value of 3.105 ± 0.01 A. Upon binding of AMPH, the RMSD value increases to 3.628 ± 0.11 A, indicating conformational changes in the enzyme upon complex formation. Since RMSDs evaluation showed ligand binding effects on conformational dynamics even at low sampling time.

**Fig. 8:**
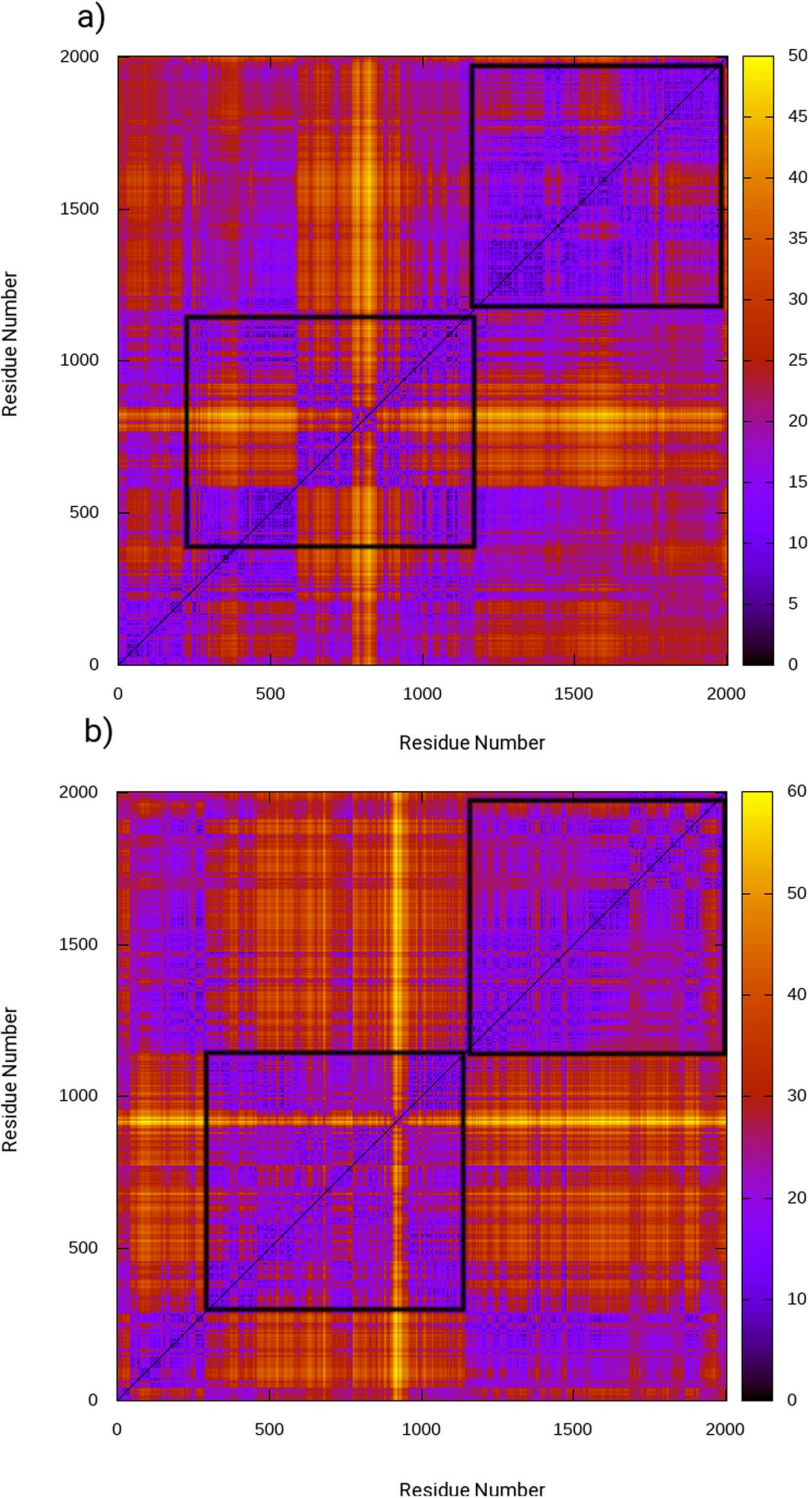
Two-dimensional Root Means Square Deviation (2D-RMSD) for **a** ligand-free molecule **b** AMPH–bound PBP2a protein

Two-dimensional root mean square deviation (2DRMSD) plots were generated for all three systems to provide more information on the conformational analysis, as shown in Fig. 8. These plots displayed a consistent trend in the maps with unique color patterns representing various types of conformations formed during the simulations. The 2D-RMS plots revealed that the ligand-bound enzyme generated a greater number of different conformations compared to the ligand-free PBPs enzyme. Furthermore, conformational change zones were identified in the encircled regions where AMPH binding occurred. Based on the 2DRMSD plots, it was determined that the AMPH-bound enzyme was more stable than the sole protein with no complex formation.

**Fig. 9:**
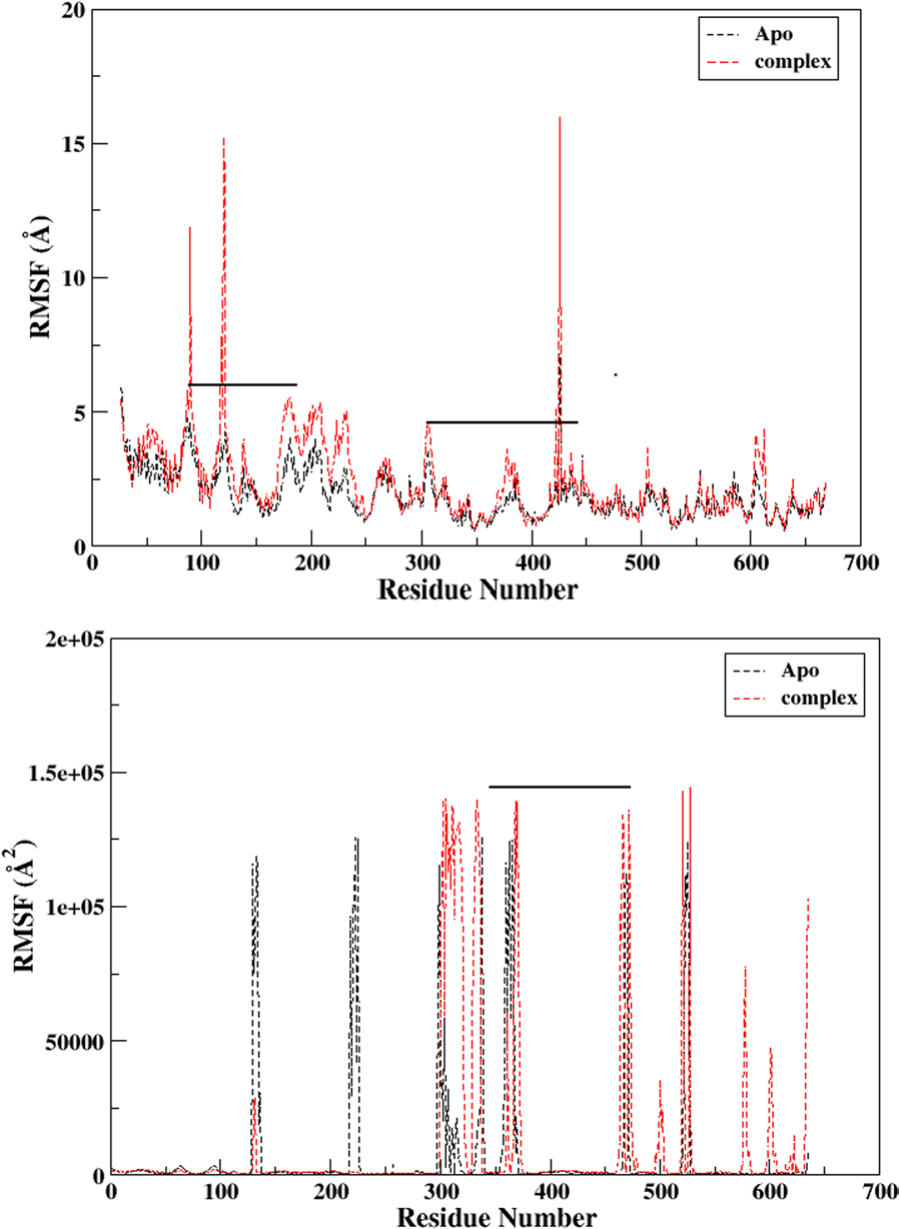
**a** Averaged root mean square fluctuation (RMSF) of only PBP2a (black) and complex showing the dynamical flexibility throughout the systems as function residue number. **b** B-factor plots for both ligand-free protein (black) and complex (red)

To investigate the impact of ligand binding on the enzyme's flexibility, RMSF plots were generated for both the ligand-free and ligand-bound states, as shown in Fig. 9. In the absence of the ligand, the enzyme exhibited significant fluctuations in the C-terminal transpeptidase domain residues ranging from 80 to 310, as well as in some regions of the non-penicillin binding domain from 435 to 605, with an average RMSF of 5.05. However, upon ligand binding, the residual dynamics of the enzyme were perturbed, particularly in the underlined region of the C-terminal active site domain from 88 to 122 and the allosteric domain from 304 to 434, with an average RMSF of 11.81. The RMSF pattern for the enzyme was distinct between the ligand-free and ligand-bound states, with fluctuations amplified in residues involved in the binding area. The averaged RMSFs were comparable to the B-factors obtained from X-ray crystallography and NMR measurements, which reflect the displacement of an atom from its mean location in the crystal structure or simulation system. The mathematical expression relating RMSF to B-factor is shown below.

6 $$B-factor=\frac{8}{3}{\pi }^{2}{RMSF}^{2}$$

The B-factor which was the averaged B-factor computed for only enzyme was found to be 345964 ± 0.99 Å which is lower than 660,013 Å that for the enzyme, thus signifying a strong impact of ligand binding on the structure and dynamics of the enzyme.

**Fig. 10:**
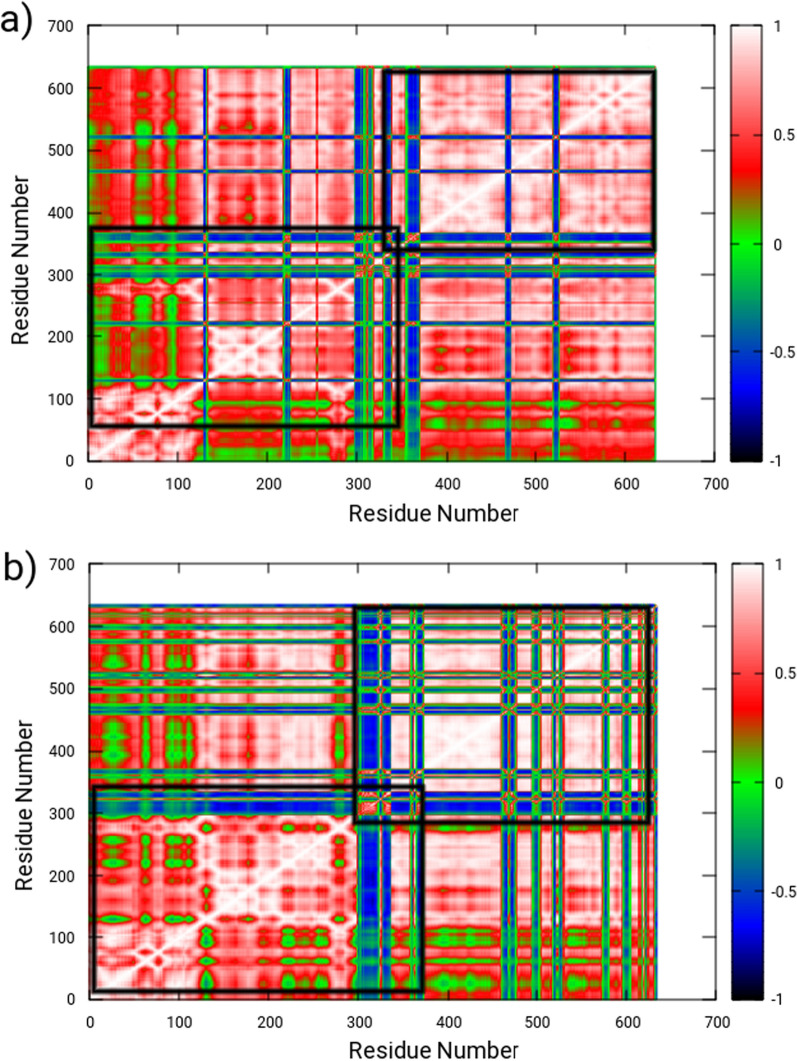
Dynamic cross correlation matrix (DCCM) maps illustrating inter-correlated motions for **a** the ligand-free, and **b** the AMPH–bound, where red contours reflect correlated movement and blue contours correspond to anti-correlated movement

In order to investigate the dynamic differences between the ligand-free and ligand-bound states of the enzyme, a dynamical cross correlation matrix (DCCM) analysis was performed. This analysis involved evaluating the covariance matrices of the Ca atoms to assess the inter-correlated movement between domains. The results were plotted in Fig. 10, where positive and negative correlation maps are represented by red and blue colors, respectively. These maps illustrate mixed correlation, with red representing correlated domain movement and blue representing anti-correlated domain movement, as a function of residue number. For the ligand-free enzyme, as shown in Fig. 10a, positively correlated movement was observed throughout in C-terminal domain of the protein from 27 to 304 amino acids whereas non-penicillin binding was found to show slight anti-correlated movements. Whereas, complex was observed to have significant anti-correlated movements in active site region and allosteric regions of the protein. This anti-correlated motion was strongly attributed to perturbations in structural dynamics.

**Fig. 11:**
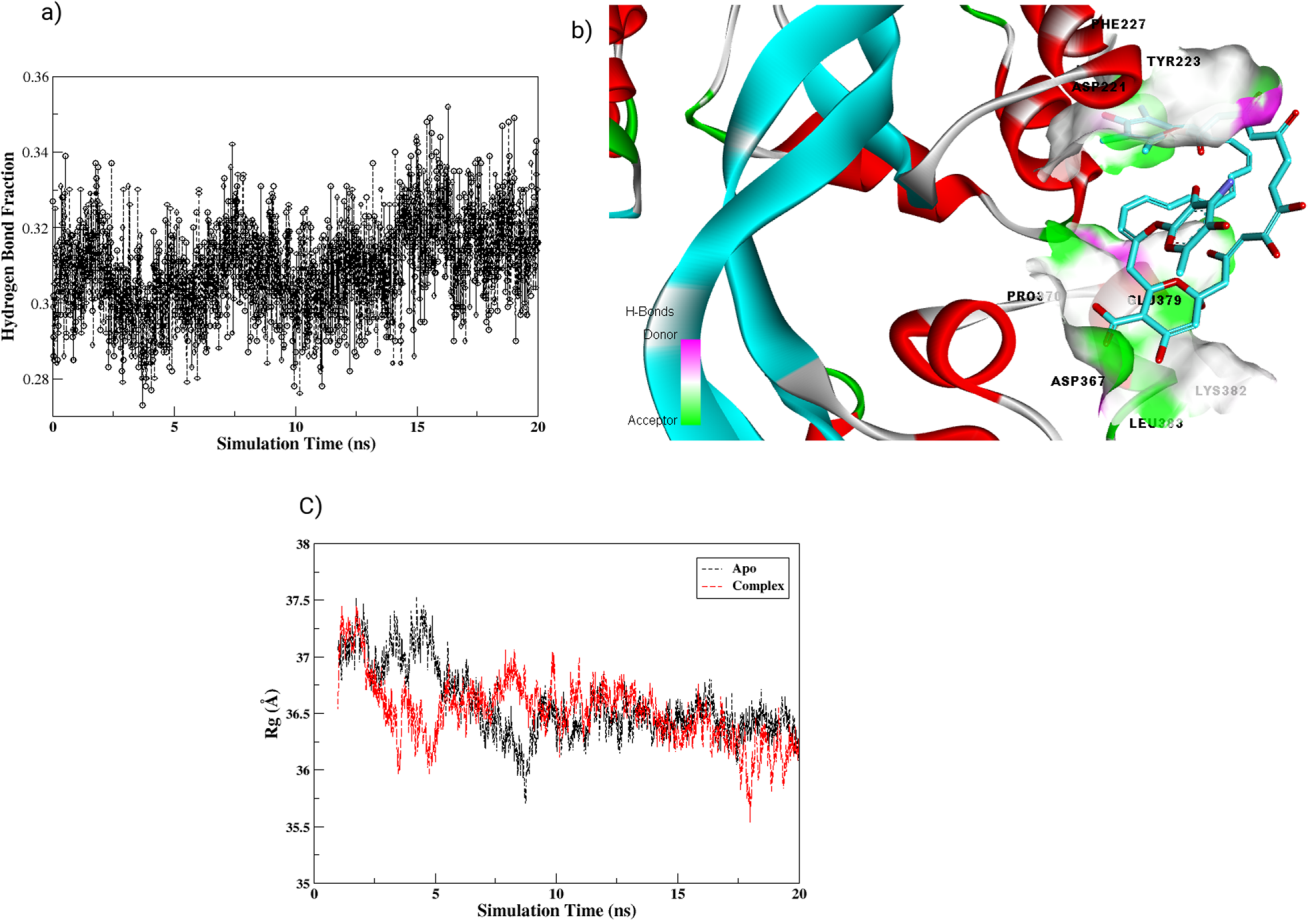
**a** Time progression of hydrogen bonds as a function of the simulation time for the AMPH bound enzyme **b** 3-D view of Hydrogen bond formation of protein amino acid residues with AMPH **c** Representation of compactness changes in protein (black) and complex (red) as radius of gyration (Rg) as function simulation

During the MD simulations, the radius of gyration (Rg) was calculated for both the protein and the complexes to assess their compactness and stability. Figure 11c shows the Rg values for the protein and PBPs-AMPH complexes, which exhibited distinct patterns throughout the simulations. The Rg of the PBPs enzyme remained relatively constant at around 36.48 ± 0.012, while the Rg of the PBPs-AMPH complex showed a slight increase to 36.57 ± 0.015 (Fig. 11a). Radius of gyration results revealed complex formation to slightly decreasing the compactness and increase the gyration of protein and thus confirms the overall disturbance in conformational dynamics of the protein. Furthermore, the interactions between the ligands and the enzyme were primarily analyzed through hydrogen bonding analysis. The number of hydrogen bond fractions was assessed as a function of simulation time, as shown in Fig. 11b and c.

The H-bond analysis for the AMPH–bound enzyme revealed the ligand binding stabilization by formation of hydrogen bonds with ASP 221, THE 227, TYR 223, PRO 370, ASP 377 and LEU.

**Fig. 12:**
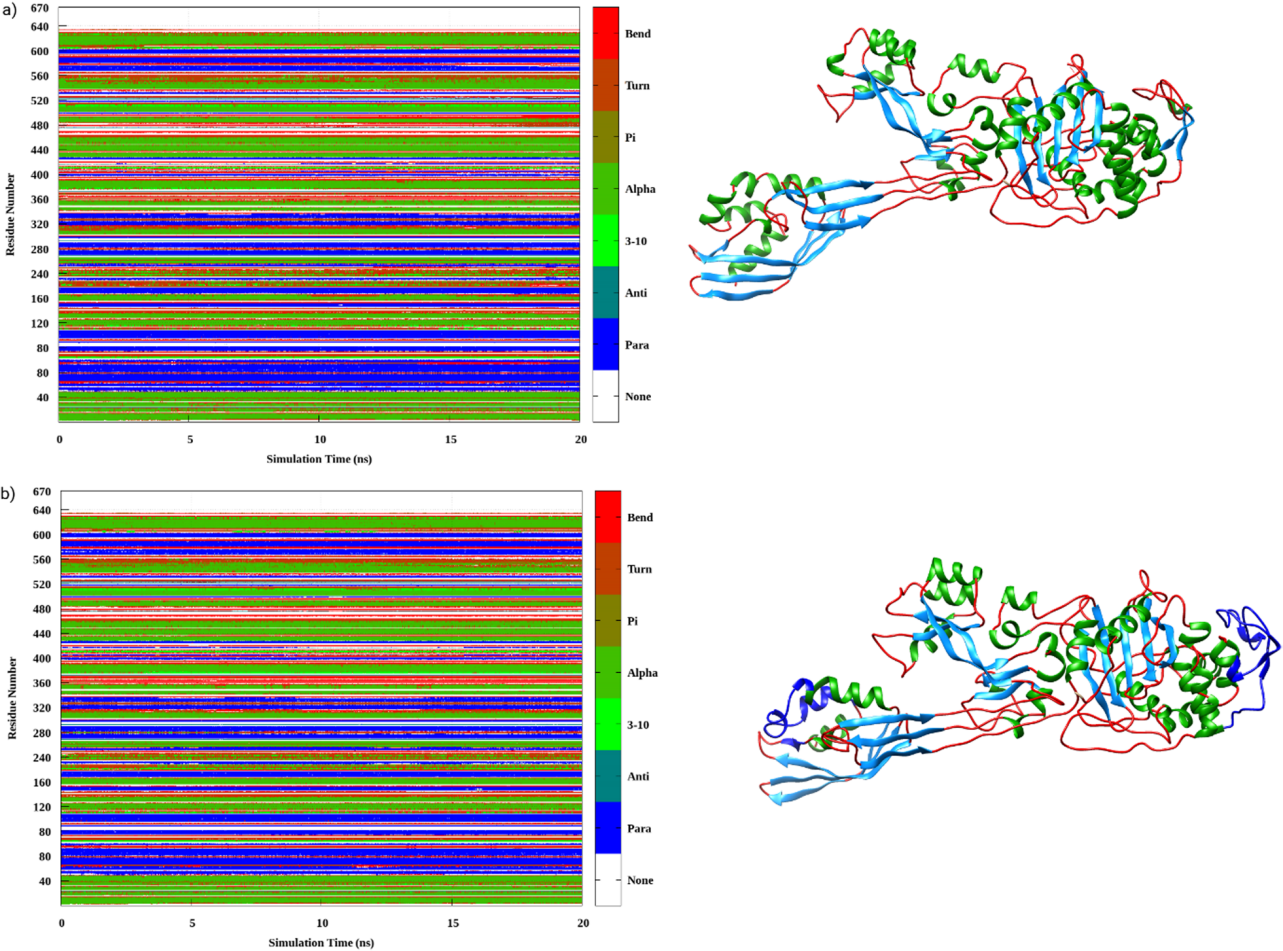
DSSP program used for Secondary structure element analysis for **a** the ligand-free, **b** the complex, respectively

The ligand binding to PBB2a caused the secondary structure elements to have significant conformational as well as structural modifications that were observed in the DSSP plots as a function of simulation time as shown in Fig. 12. A detailed assessment of the DSSP plots revealed notable conformational perturbations in the enzymatic structure upon complexation like amino acid residues from 220 to 240 and 310 to 320 (marked with color changes Fig. 12b) were to have conformational perturbations. In the non-penicillin binding domain amino acids residues from 530 to 560 were also observed to be effected by ligand binding.

**Fig. 13:**
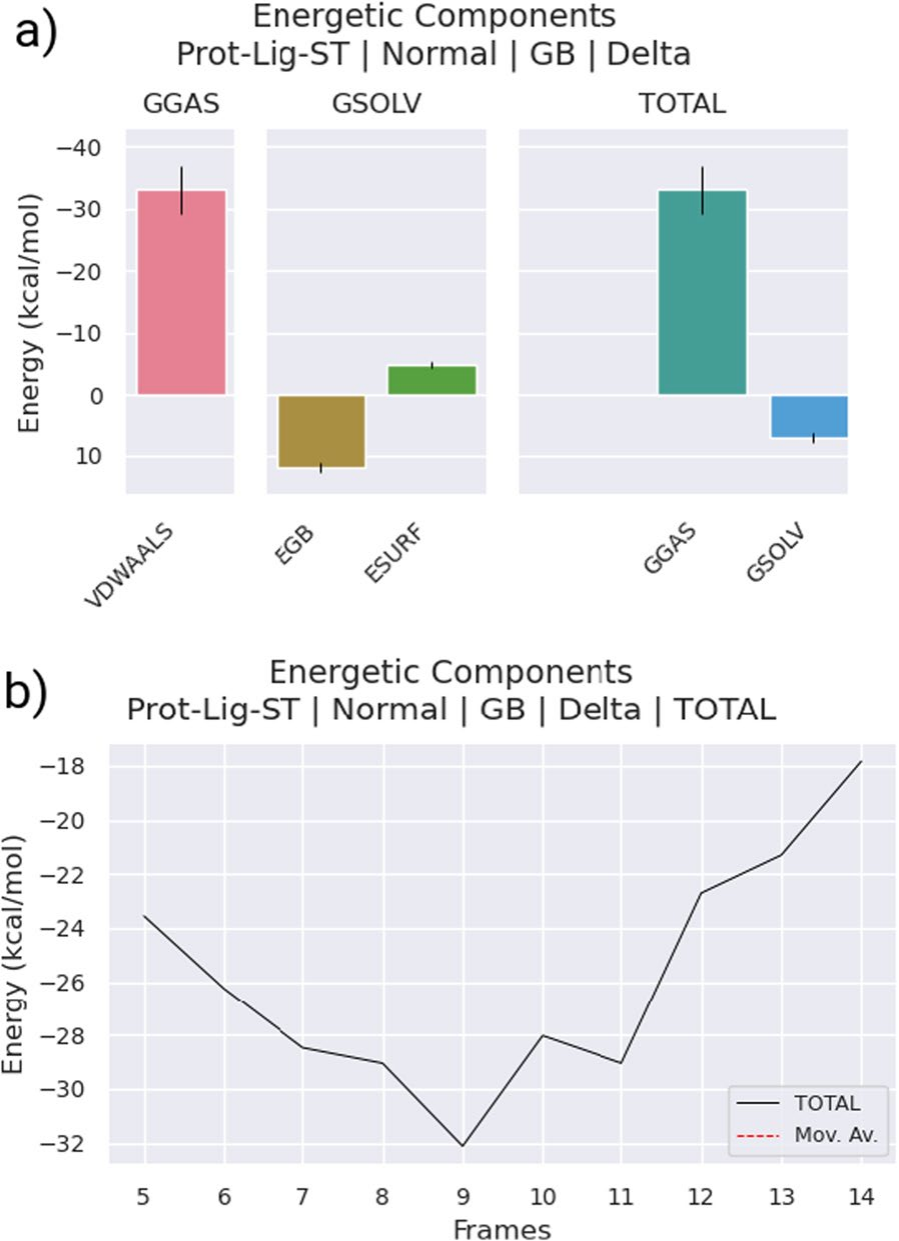
**a** MMPSA scheme of the complex representing each contributing energetics components **b** Total energy count as function of number of frames from equilibrated parts of simulated trajectory

The MM/PBSA method was utilized to calculate the binding free energy between PBPs and AMPH, and to gain more detailed information about their interactions. Table 3 presents a comprehensive analysis of the binding free energies and energy components of the complexes. The results show that AMPH had a negative binding energy and the lowest binding energy value of −25 kJ/mol. Four energy components, including van der Waals (DEvdw), electrostatic (DEele), polar solvation energy (DGpol), and nonpolar interactions (DGnonpol), were calculated to assess the major contributions of each interaction term in the binding process. Results from Table 3 and Fig. 13 indicate that DEvdw and DEele played significant roles in the formation of the complexes. Specifically, van der Waals interactions had a significant impact on the binding of AMPH to the chosen compounds due to the significant non-covalent interactions formed between the two. Additionally, the non-polar interaction energies (DEvdw DGnonpol) and polar interaction energies (DEele DGpol) were calculated, with results indicating that favorable non-polar interactions primarily mediated the interactions between the AMPH binding pocket and the chosen chemicals (−25.84 kJ/mol).

**Table 3:** MMPBSA calculations of the simulated complex

S.No	Energies	Amphotericin B AMPH
1	Vander Waals ${\Delta G}_{vdw}$	−33.05
2	Surface ${\Delta G}_{SURF}$	−3.124
3	Solvation ${\Delta G}_{SOLV}$	7.22
4	Gaseous ${\Delta G}_{GAS}$	−33.05
5	Total binding energy ${\Delta G}_{binding}$	−25.84

## Conclusion

In this study, Antibacterial potential of Amphotericin B (AMPH) was discovered using applying antimicrobial screening and molecular docking mode of action analysis and structural dynamics evaluations via Molecular dynamics simulations. Antimicrobial screening revealed significant antibacterial potential of the compound showing reasonable MIC (16 µg/ml) and MBC (32 µg/ml). Molecular docking simulations then confirmed the significant potential of ligand showing its strong binding with protein in both active site, C terminal and non-penicillin binding regions. MD simulations revealed remarkable high stability of the main compound–AMPH complex. Interestingly, these compounds showed the ability to bind to the both C, terminal, active site domain as well as non-penicillin binding domain, responsible for allosteric regulations. Therefore, it can be considered as the potent antibacterial agent for combating pathogenic microbial ailments generated by especially β-lactams resistant microbial strains. Unlike the earlier studies which have reported only the antifungal potential of the compound-Amphotericin B, this is the first study ever reporting antibacterial potential of the selected compound, with strong binding mode to the PBP2a protein at both active sites and allosteric regions. Furthermore, assessment hydrogen bond formation and free energy of binding revealed the potential of compound to be supported by hydrophobic and hydrophilic interactions. Based on the outcomes, this study can set a bench mark in future for designing inhibitor molecules against the PBP2a enzyme, and can be streamlined in an extensive perception to design potential drug against infectious diseases caused by β-lactams resistant bacteria.

## Data Availability

All data generated or analysed during this study are included in this published article.
